# Cardiac Optical Coherence Tomography: History, Current Status, and Perspective

**DOI:** 10.1016/j.jacasi.2023.10.001

**Published:** 2023-11-28

**Authors:** Taishi Yonetsu, Ik-Kyung Jang

**Affiliations:** a Department of Cardiovascular Medicine, Tokyo Medical and Dental University, Tokyo, Japan; b Cardiology Division, Massachusetts General Hospital, Harvard Medical School, Boston, USA

**Keywords:** acute coronary syndrome, optical coherence tomography, percutaneous coronary intervention, plaque

## Abstract

For more than 2 decades since the first imaging procedure was performed in a living patient, intravascular optical coherence tomography (OCT), with its unprecedented image resolution, has made significant contributions to cardiovascular medicine in the realms of vascular biology research and percutaneous coronary intervention. OCT has contributed to a better understanding of vascular biology by providing insights into the pathobiology of atherosclerosis, including plaque phenotypes and the underlying mechanisms of acute coronary syndromes such as plaque erosion, neoatherosclerosis, stent thrombosis, and myocardial infarction with nonobstructive coronary arteries. Moreover, OCT has been used as an adjunctive imaging tool to angiography for the guidance of percutaneous coronary intervention procedures to optimize outcomes. However, broader application of OCT has faced challenges, including subjective interpretation of the images and insufficient clinical outcome data. Future developments including artificial intelligence–assisted interpretation, multimodality catheters, and micro-OCT, as well as large prospective outcome studies could broaden the impact of OCT on cardiovascular medicine.

## Highlights

• Intravascular OCT has been used for 2 major purposes: vascular biology research and PCI guidance.
• OCT provides nearly 10 times higher spatial resolution as IVUS, enabling detailed observation of vessel structures.
• OCT-guided PCI has been shown to provide noninferior outcomes compared with IVUS-guided PCI.
• Future developments are required to overcome the limitations of the current OCT systems including subjective image interpretation.

## History of Intravascular Optical Coherence Tomography

**Figure 1:**
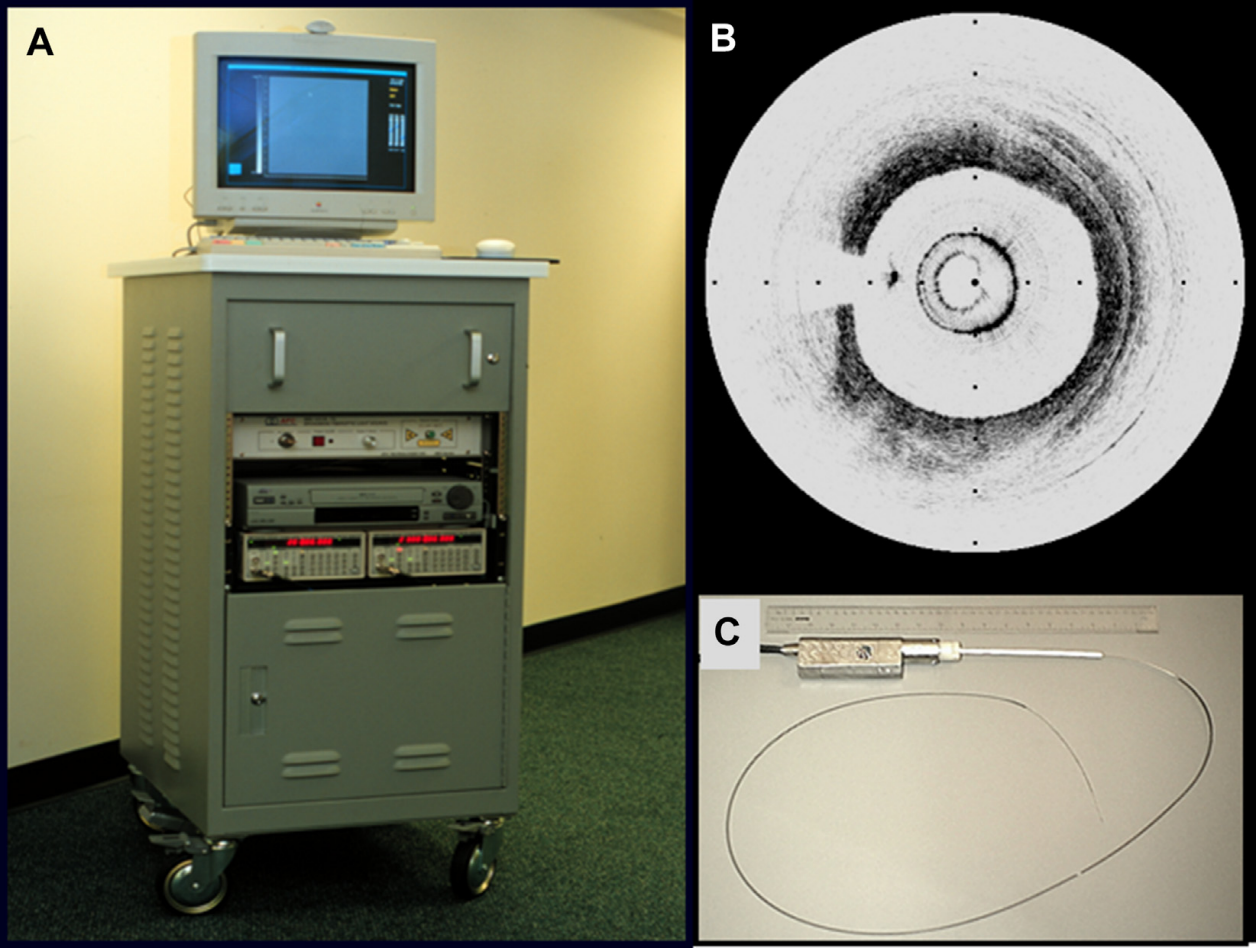
Prototype Console (A) Prototype OCT console built at Wellman Laboratory of Photomedicine, Massachusetts General Hospital. (B) Image was acquired by flushing saline distal to the occlusion balloon. (C) Prototype catheter was built by replacing the transducer core of a commercial intravascular ultrasound catheter with an optical fiber. Reprinted with permission from Yonetsu et al.^87^ OCT = optical coherence tomography.

**Figure 2:**
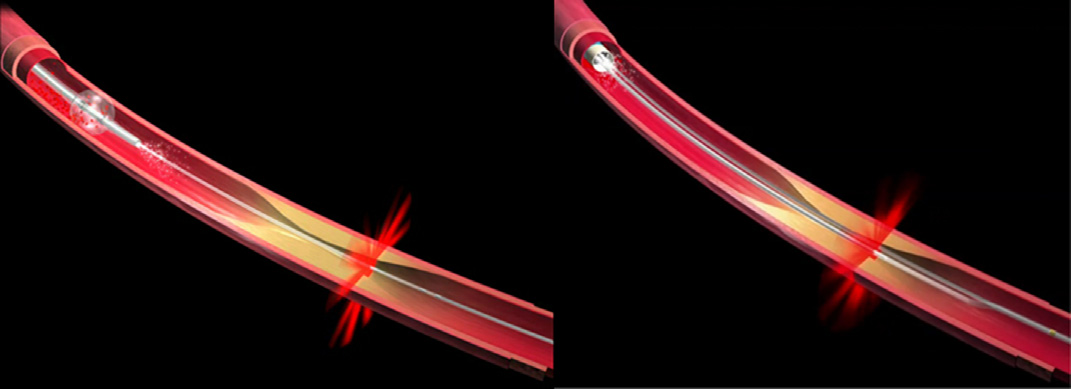
Time-Domain and Frequency-Domain OCT Owing to the limited pull-back speed of time-domain OCT system, it took ≥30 seconds to image a 50-mm segment. For the elimination of blood during the OCT image acquisition, an occlusion balloon was placed proximal to the lesion and saline was injected from the tip of the occlusion balloon (left). Frequency-domain system enabled faster pull-back and a 75-mm segment can be imaged in approximately 2 seconds, which does not require occlusion balloon to eliminate blood (right). Abbreviation as in Figure 1.

In 1998, 6 years after the invention of optical coherence tomography (OCT) technology by Professor Fujimoto at the Massachusetts Institute of Technology (MIT),^1^ the Cardiac OCT group was established at Massachusetts General Hospital (MGH) (Central Illustration), which consisted of 3 members: 2 scientists from MIT, Brett Bouma and Guillermo Tearney, and a cardiologist, Ik-Kyung (IK) Jang. Their first endeavor was to validate the OCT findings of various plaque types against histology. By analyzing 357 human vessel specimens (162 aortas, 105 carotids, and 90 coronary arteries) from 90 cadavers, they reported in 2002 that OCT could accurately characterize plaque types with sensitivities and specificities of 71% to 79% and 97% to 98% for fibrous plaque, 95% to 96% and 97% for calcified plaque, and 90% to 94% and 90% to 92% for lipid-rich plaques.^2^ During the validation study, the cardiac OCT group continued to develop a clinically viable catheter-based OCT system at the Wellman Laboratory of Photomedicine at MGH. The prototype OCT console was heavy and bulky, requiring adjustment of various parameters after each image acquisition (Figure 1). The catheters were built by replacing the transducer core of a commercial intravascular ultrasound (IVUS) catheter with an optical fiber. After preclinical studies to validate feasibility and safety, Jang conducted the first-in-man procedure, enrolling patients undergoing percutaneous coronary intervention (PCI). This first study was done in Korea in collaboration with Dr Seung-Jung Park at Asan Medical Center and Dr Ki-Bae Seung at Catholic University Hospital. Because the prototype machine was not stable, several staff flew ahead with the machine. When they checked the machine as soon as it arrived in Korea, they expectedly found some parts were damaged. The second team brought parts to replace those that were damaged. Like any other new procedures, unexpected complications could occur, but complications were of particular importance because OCT is an intracoronary procedure. A critical complication not only would have harmed the patient, but it could have also jeopardized the future of cardiac OCT. Fortunately, intracoronary OCT imaging in living patients was safely performed in 2000 without any complications. In 2002, the first report on plaque characteristics of culprit lesion in patients with acute myocardial infarction (AMI), acute coronary syndromes (ACS), and stable angina was published, confirming the previous pathology studies.^3^ Since the prototype OCT system was not equipped with automated pull-back, the catheter was manually pulled back to the region of interest and saline was injected to remove blood to acquire several images for each plaque. After the research use of the prototype OCT system, a commercial system was developed by LightLab Imaging Inc. in Westford, Massachusetts, as an MIT startup in a joint venture with Carl Zeiss Meditec. The first commercially available intravascular OCT system used time-domain (TD) detection (TD-OCT). The TD-OCT catheter had an outer diameter of 0.019-inch, containing a 0.006-inch optic fiber, that appeared like a guide wire (ImageWire LightLab Inc). TD-OCT provided a slow image acquisition at 15 to 20 frames/second (200-240 axial scans per frame), resulting in a maximum pull-back speed of 2 mm/second, which required proximal occlusion of the blood flow with a dedicated balloon and saline infusion from the tip of the occlusion balloon (Figure 2).^4^

**Central Illustration:**
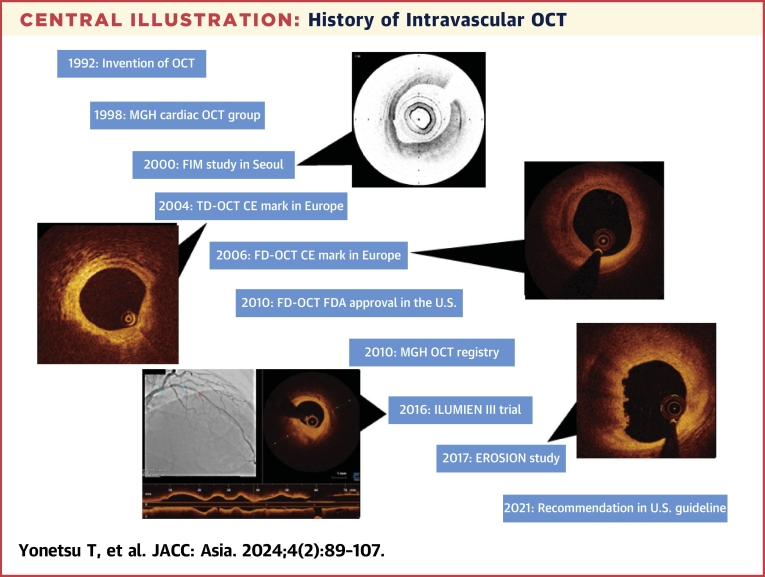
History of Intravascular OCT Timeline of intravascular OCT development and landmarks is summarized. For 20 years since its invention at the Massachusetts Institute of Technology, intravascular OCT has emerged as an imaging tool for coronary arteries, enhancing our understanding of atherosclerosis and aiding percutaneous coronary interventions procedures. CE = Communauté Européenne; FDA = U.S. Food and Drug Administration; FD-OCT = frequency-domain optical coherence tomography; FIM = first-in-man; MGH = Massachusetts General Hospital; OCT = optical coherence tomography; TD-OCT = time-domain optical coherence tomography; U.S. = United States.

With the advent of commercially available equipment, multiple groups conducted validation studies to show the accuracy and feasibility of a commercial intravascular OCT imaging system. Kume et al^5^ examined 35 lipid-rich plaques from 38 human cadavers and evaluated the correlation between the OCT-based measurement of fibrous cap on lipid-laden plaque and histology examination and reported an excellent correlation between OCT and histology measurements of fibrous cap (R = 0.90; *P* < 0.001). The same group reported the categorization of intraluminal thrombus by OCT. In the examination of 108 coronary arterial segments from 40 cadavers, red thrombus was characterized by high-backscattering protrusion with signal-free shadowing, whereas white thrombus was characterized by low-backscattering projection.^6^ TD-OCT acquired the CE mark in Europe in 2004; however, because of the need for proximal occlusion, the U.S. Food and Drug Administration did not approve it. Subsequently, in 2006, frequency-domain (FD)-OCT was introduced, which revolutionized intracoronary imaging. Unlike TD-OCT, FD-OCT was user friendly and provided much faster image acquisition enabling faster pull-back speeds of 20 mm/second. With FD-OCT, >50 mm could be imaged within 3 seconds. This nonocclusive image acquisition facilitated the use of OCT and, finally in 2010, the U.S. Food and Drug Administration approved intravascular OCT for clinical use.

In the same year, the MGH OCT Registry was launched. Data have been collected at 21 sites from 6 countries around the world. This registry included a broad spectrum of patients including those with ACS and chronic coronary syndrome, those who underwent 3 vessel OCT imaging, and those with previously implanted stents. Since the OCT registry was launched, >40 fellows from around the globe have been trained at the Cardiology Laboratory for Integrated Physiology and Imaging at MGH in Boston, Massachusetts.

### OCT’s contributions to cardiovascular medicine

There are 2 primary applications of intravascular OCT imaging: in vivo vascular biology research and PCI optimization. Intravascular OCT provides high-resolution images of coronary arteries with a 10- to 20-μm spatial resolution, nearly 10 times higher than that of IVUS examination (Table 1). Moreover, the greater signal-to-noise ratio and the ability to differentiate plaque components enables detailed visualization of vascular structures, often referred to as an optical biopsy. These benefits have facilitated the use of OCT in research to study vascular biology, which previously could not be done by existing modalities, and during PCI procedures where OCT can accurately assess vessel size and stent apposition. Given that the strength of OCT lies in visualizing microstructures related to plaque vulnerability, such as lipid-rich plaque, fibrous cap thickness, macrophage accumulation, and intraluminal thrombus, OCT drew attention as an emerging imaging modality to evaluate plaque vulnerability in vivo. Before the commercial OCT system became available, Jang et al reported higher prevalence of lipid-rich plaque, thinner fibrous cap, and more frequent thin-cap fibroatheroma (TCFA) defined as having ≥2 quadrants of lipid and fibrous cap <65 μm in patients with AMI compared with non–ST-segment elevation MI (STEMI) or patients with stable angina patients.^7^ This report marked the beginning of in vivo vascular biology research using OCT. In terms of visualization of the culprit lesions of AMI, Kubo et al^8^ reported that OCT is superior to IVUS examination and coronary angioscopy for detection of plaque rupture with thrombus.

**Table 1:** Comparison Between Intravascular Ultrasound Examination and OCT in the Identification of Plaque Morphology

	Intravascular Ultrasound (Grayscale)	OCT
Spatial resolution	120-200 μm	10-20 μm
Pull-back speed	0.5-10 mm/s	18-36 mm/s
Tissue penetration	8 mm	1-2 mm
Frame rate	30 frame/s	180 frame/s
Elimination of blood	Not required	Required
Lipid core	+	+++
Fibrous cap	+	+++
Remodeling	+++	+
Calcium	++	+++
Thrombus	+	++
Macrophage		+

OCT = optical coherence tomography.

OCT has been tested for its use to optimize PCI in clinical practice. OCT-guided PCI comprises 3 phases: vessel morphological assessment, vessel size determination, and stent optimization. Before interventional procedures, OCT can provide detailed information about plaque characteristics, including the presence and extent of lipid-rich plaques and calcification. Plaques containing a substantial lipid component are susceptible to distal embolization, potentially resulting in slow flow phenomenon or periprocedural MI, complications that can be predicted with baseline OCT images.^9^^,^^10^ Lee et al^10^ observed 131 patients undergoing PCI and reported that TCFA was independently associated with an increased risk of periprocedural MI. Although the usefulness of distal protection devices remains contentious, predicting periprocedural MI may aid in devising PCI strategies to circumvent distal embolization. Moreover, one advantage of OCT over IVUS examination is its superior delineation of calcified plaques, enabling the quantification of calcium arc and thickness.^11^^,^^12^ A study revealed that calcium thickness predicts the vessel’s crackability by balloon angioplasty,^13^ potentially informing the decision to use debulking devices, such as rotational or orbital atherectomy, or intravascular lithotripsy to achieve sufficient stent expansion. Apart from plaque characterization, OCT enables precise measurements of vessel size and lesion length, facilitating the selection of appropriate coronary stents. After stent implantation, OCT is adept at detecting minor complications like intimal dissection or stent malposition,^4^^,^^14^ in addition to offering quantitative measurements of stent expansion. Because insufficient stent expansion is a critical predictor of stent failure, further postdilatation may be performed to optimize stent expansion. Before the integration of OCT into clinical practice, angiograms and IVUS examinations were used to guide optimal stent implantation. However, the superior image quality of OCT may potentially supersede these modalities. Consequently, OCT is expected to provide indispensable information on vascular biology and to assist in PCI guidance for physicians. The following sections offer a synopsis of the history and current state of OCT research in these 2 directions.

## Current status

In the previous version of the American College of Cardiology/American Heart Association/Society for Cardiovascular Angiography and Interventions PCI guidelines published in 2011,^15^ OCT technology was mentioned without specific recommendations, whereas IVUS examination received Class IIa recommendations for ambiguous left main disease and stent restenosis. Owing to the lack of guideline-based recommendations, intracoronary OCT use remained minimal in the United States for a decade. In fact, a large database from the Healthcare Cost and Utilization Project’s National Inpatient Sample, encompassing 3 million cases of coronary angiography, reported that OCT was used in a mere 0.3% of PCI procedures performed in 2014.^16^ Similarly, in Europe, OCT use was reported at only 1.3% of PCI cases between 2005 and 2015, although the rate slightly increased during that period.^17^ In contrast, OCT penetration rates were higher in Asian countries, particularly in Japan, where the national guidelines granted Class I recommendations for the use of intravascular imaging for stent optimization, and imaging modalities were reimbursed by national health insurance.^18^ According to the annual report from the Japanese Circulation Society, OCT was used in 29,700 of 255,416 (11.6%) PCI procedures, and IVUS examination was used in approximately 80% of cases in 2014. These data indicate that >90% of PCI procedures were guided by intravascular imaging. In general, intravascular imaging techniques are used more commonly in Asian countries, including Japan, Korea, and China. However, recent data on the exact rate of OCT use in PCI have not been reported in the literature. Thus, although OCT-guided PCI rates varied among countries, the overall penetration rate remained limited. Over the last decade, a greater number of randomized controlled trials were conducted to evaluate the advantages of imaging-guided PCI, primarily based on IVUS. In the early 2010s, small-scale studies failed to demonstrate the benefits of intravascular imaging modalities^19^^,^^20^; however, recent large trials have reported superior clinical outcomes and lower stent failure rates with intravascular imaging-guided PCI, compared with angiography-guided PCI.^21^, ^22^, ^23^, ^24^ In the context of OCT, several randomized trials have shown comparable acute results and long-term outcomes after OCT-guided PCI, as opposed to IVUS-guided PCI, with superior stent expansion compared with angiography-guided PCI.^25^, ^26^, ^27^, ^28^ Considering the significant advancements in the field of PCI, such as coronary stents, PCI procedures, pharmacological interventions, and imaging modalities, the American College of Cardiology/American Heart Association/Society for Cardiovascular Angiography and Interventions guidelines were updated in 2021. This update consolidated several guidelines for PCI, coronary artery bypass grafting, primary PCI for STEMI, and non-ST-segment elevation (NSTE)-ACS. IVUS-guided PCI received Class IIa recommendations for complex PCI and left main disease, whereas OCT was recommended as an alternative modality to IVUS examination, except for ostial lesions.^29^ This update may promote the use of OCT as a complementary imaging modality to angiography for PCI.

## OCT as a Clinical Tool

### PCI optimization

**Figure 3:**
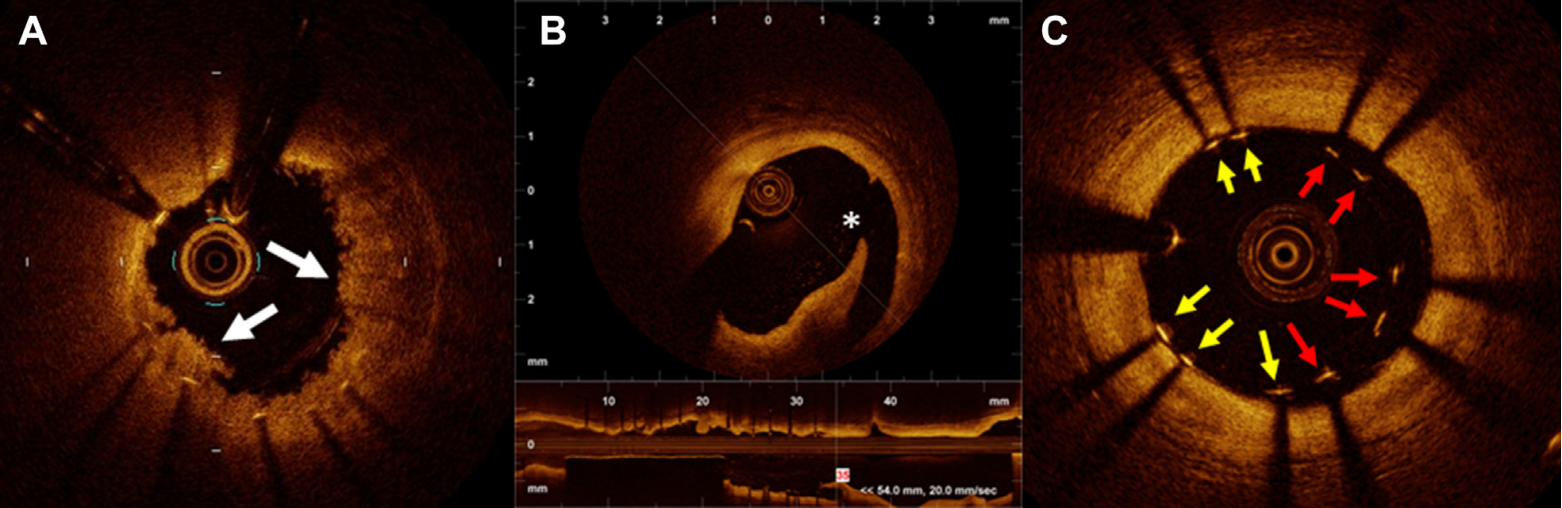
Complications After Stent Implantation (A) Irregular protrusion is defined as protruding material with irregular surface (arrows) inside the stent. (B) Stent edge dissection is identified as a tear of intimal tissue (asterisk) at the edge of implanted stent in both cross-sectional view and longitudinal view. (C) Endoluminal surface of stent strut is visualized as a linear and bright signal accompanied by a shadow behind the signal (arrows). When the distance between the signal and lumen surface exceed the thickness of stent strut, the strut is considered malapposed (red arrows).

**Figure 4:**
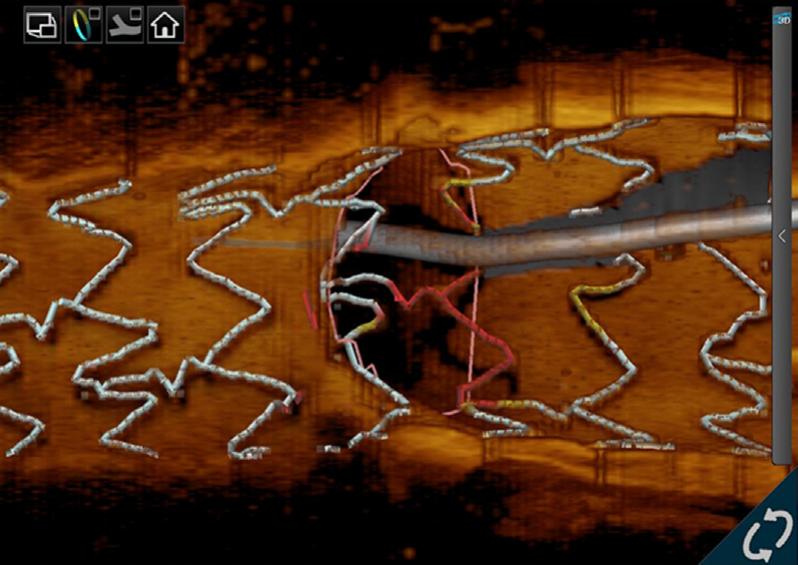
3-Dimensional Reconstruction of Stent Structure 3-dimensional (3D) reconstruction of OCT images provides visualization of precise stent structure in relation to the jailed side branch. Location of a guidewire recrossed into the side branch can be recognized on the 3D image. Abbreviation as in Figure 1.

For intravascular OCT to gain acceptance as an adjunctive imaging device for PCI, it needs to provide data that its use would improve outcomes. Thus, clinical outcomes after PCI have become a research focus in this regard. Previous studies have reported several predictors of stent failure as assessed by OCT.^30^, ^31^, ^32^, ^33^, ^34^ The minimum stent area (MSA) has been one of the most significant predictors of stent failure since the bare metal stent era.^35^ After the advent of drug-eluting stents, multiple IVUS studies advocated various cut-off thresholds of MSA to avoid stent failure: 5.0 mm^2^ for sirolimus-eluting stents^36^ and 5.7 mm^2^ for paclitaxel-eluting stents.^37^ These thresholds have been used for procedural endpoints in IVUS-guided PCI. However, OCT underestimates the lumen area in comparison with IVUS by approximately 10%.^38^ This discrepancy must be taken into consideration when adopting IVUS-derived thresholds for OCT-guided PCI. From the data of MGH OCT registry, including 900 lesions in 786 patients, Soeda et al^30^ investigated the OCT-derived predictors of device-oriented cardiac events and found that the best cut-off threshold for MSA was 5.0 mm^2^. This paper also reported that irregular protrusion inside the implanted stent was an independent predictor of device-oriented cardiac events. In addition to MSA and irregular protrusion, edge dissection^32^^,^^33^ and stent malapposition^39^^,^^40^ have been reported as potential predictors of subsequent stent failure (Figure 3). A significant advantage of OCT in PCI is the 3-dimensional reconstruction of the implanted stent structure over bifurcated lesions.^41^ Recent OCT consoles are equipped with 3-dimensional reconstruction software, facilitating the identification of stent struts, link connections, and guidewire recrossing positions (Figure 4). Previous studies indicated that the likelihood of incomplete stent apposition after kissing balloon dilatation to the bifurcation could be predicted by the type of link connection of stent strut and the position of the recrossed wire.^42^ This information could contribute to an optimization of stent implantation in bifurcation lesions, potentially decreasing the risk of late complications, including stent thrombosis. Calcified lesions present a considerable challenge in the optimization of PCI, often preventing sufficient stent expansion and increasing the risk of stent failure.^43^ Unlike IVUS examination, which exhibits acoustic shadows behind the calcification, OCT depicts calcification as a clearly delineated, signal-poor region, enabling the measurements of the thickness of calcific plates.^44^ In cases of a thick and circumferential calcification, balloon angioplasty is not effective to dilate the vessel.^13^ Instead, debulking devices such as rotational atherectomy, orbital atherectomy, or lithotripsy should be considered to achieve sufficient stent expansion.

### Clinical studies

**Figure 5:**
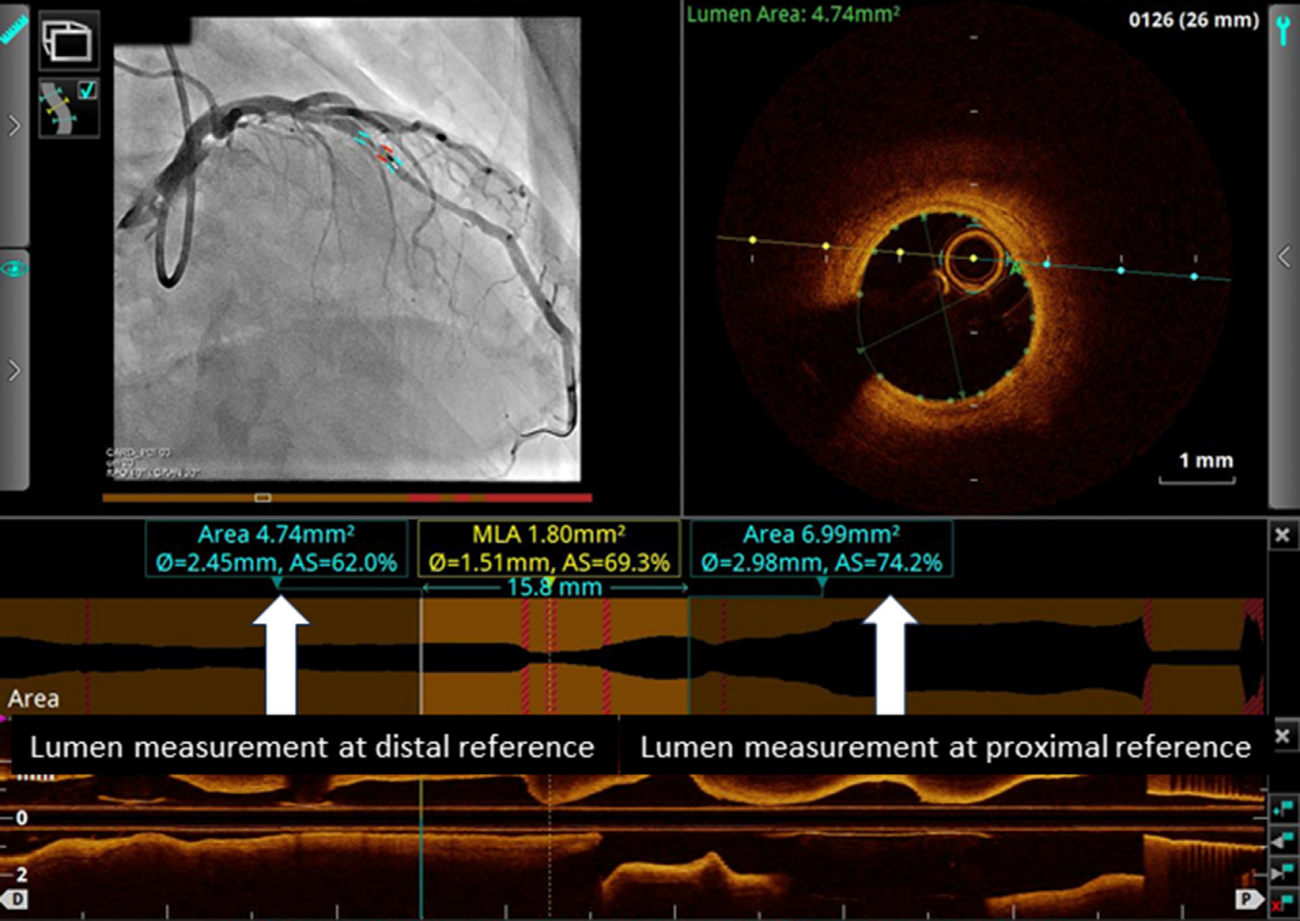
Measurement of Lumen Profile and Selection of Stent Size Equipped software automatically traces the lumen contour, and the lumen profile is displayed in the middle. The ILUMIEN III study proposed a standard protocol of stent selection: stent size was determined by the smallest external elastic lamina diameter of the proximal or distal reference site, rounding it down to the nearest 0.25 mm when those were visible; otherwise, proximal and distal lumen diameters were used. In the OPINION study, the stent diameter was determined as 0 to 0.25 mm greater than mean lumen diameter at distal reference site. In the present case, lumen diameter at distal reference was 2.45 mm (white arrows), and the operator implanted a 2.75-mm stent according to the OPINION protocol.

**Figure 6:**
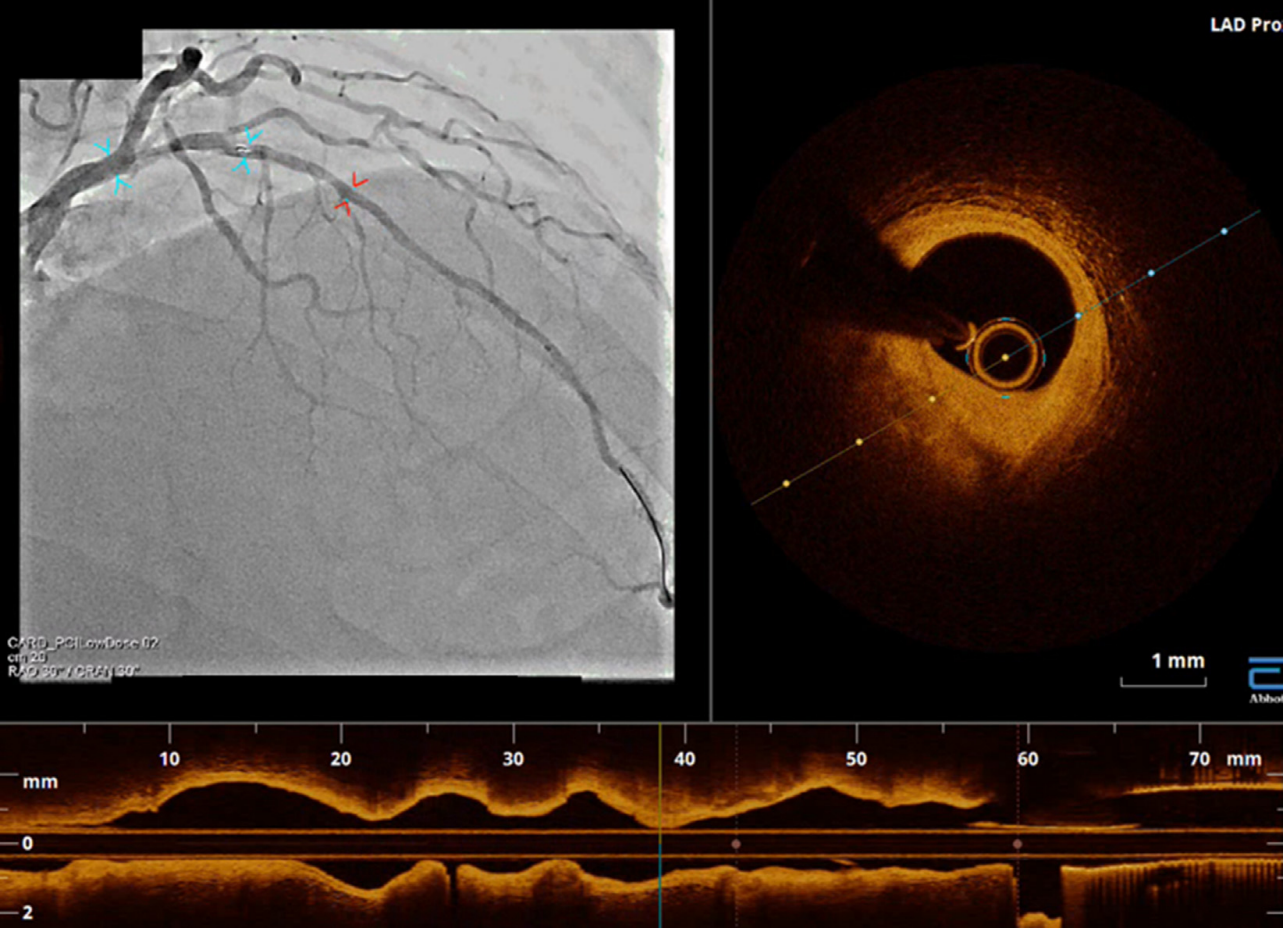
Angio-Coregistration in a Contemporary Console Simultaneous acquisition of angiograms and optical coherence tomography images enables precise location of the acquired images.

The clinical impact of OCT-guided PCI has been tested in several prospective studies. The ILUMIEN I (Observational Study of Optical Coherence Tomography in Patients Undergoing Fractional Flow Reserve and Percutaneous Coronary Intervention) study was a single-arm prospective observational study which investigated the impact of OCT assessment on physician decision-making.^45^ This study found that 57% of strategies were changed by examining baseline plaque morphology with OCT before PCI, and additional procedures were performed after OCT assessment of implanted stents in 27% of cases. This finding highlights that OCT assessment may change PCI strategy when added to angiography-guided PCI. Since OCT has been introduced, it has been compared with IVUS examination in various aspects. The benefits of OCT encompass high-resolution imaging, the capacity to distinguish lipid-containing plaque, and the visualization of coronary artery microstructures. This allows a meticulous evaluation of atherosclerosis. IVUS examination, however, facilitates the visualization of the entire vessel, thereby enabling the assessment of vascular remodeling and plaque burden. Beside their distinctive imaging features, OCT imaging requires the clearance of blood from the vessel lumen during the image acquisition process, which impedes the use of OCT in lesions involving the ostium or severe stenosis. Given these relative strengths and weaknesses, both modalities have been the subject of comparison in previous clinical studies (Table 2). The ILUMIEN II study was a post hoc analysis of the ILUMIEN I and ADAPT-DES (Assessment of Dual Antiplatelet Therapy With Drug-Eluting Stents) studies, comparing acute results and stent expansion between OCT-guided PCI in ILUMIEN I and IVUS-guided PCI in the ADAPT-DES study. The study revealed nearly identical stent expansion between the 2 arms and more frequent detection of minor complications with OCT such as malapposition, tissue protrusion, or stent edge dissection.^25^ This study suggested that OCT might serve as an alternative imaging modality to IVUS examination for PCI guidance. Building on previous trials, the ILUMIEN III trial was conducted to prospectively compare acute results and 30-day adverse events among IVUS-, OCT-, and angio-guided PCI. Ali et al^27^ reported that OCT-guided PCI provided a similar mean minimal stent area to that of IVUS-guided PCI, and there were no significant differences in the rates of adverse events. This trial prespecified standard procedures for OCT-guided PCI. Specifically, stent size was determined by the smallest external elastic lamina diameter of the proximal or distal reference site, rounding it down to the nearest 0.25 mm when those were visible; otherwise, proximal and distal lumen diameters were used (Figure 5). Stent length was decided to cover the proximal and distal reference sites, which were defined by the automated lumen detection feature of the console identifying the largest lumen sites along the lesion. Postdilatation was performed to achieve a minimal lumen area of >90% of the reference sites. Furthermore, advancements in software, including angiogram coregistration and automated lumen detection features (Figure 6), have provided useful information seamlessly during PCI. Contemporary software installed in the console enables on-site determination of stent size and length during PCI procedures. The OPINION (Optical Frequency Domain Imaging vs. Intravascular Ultrasound in Percutaneous Coronary Intervention) trial was a randomized controlled trial comparing OCT-guided and IVUS-guided PCI in terms of clinical outcomes at 1 year, using the OCT system produced by Terumo, called optical frequency domain imaging (OFDI). Although OFDI is based on the same FD-OCT technology as FD-OCT produced by LightLab/St. Jude/Abbott, image acquisition rates and pull-back speeds are different. Kubo et al^26^^,^^46^ reported that the adverse event rate was comparable between IVUS-guided PCI and OFDI-guided PCI, demonstrating the noninferiority of OFDI guidance. OCT-guided PCI was also tested in the ACS setting. The DOCTORS (Does Optical Coherence Tomography Optimize Results of Stenting) study was a randomized controlled trial comparing OCT- and angio-guided PCI for the primary endpoint, defined as the fractional flow reserve (FFR) value immediately after PCI, enrolling 240 patients with NSTE-ACS.^28^ The FFR value and angiographic diameter stenosis were better in the OCT-guided arm than in the counterpart. This result highlighted the potential superiority of OCT-guided PCI in patients with NSTE-ACS over angio-guided PCI. Another trial, OCTACS (Optical Coherence Tomography Guided Percutaneous Coronary Intervention With Nobori Stent Implantation in Patients With Non–ST-Segment–Elevation Myocardial Infarction), compared OCT- and angio-guided PCI regarding the rate of malapposed struts and uncovered struts of biolimus-eluting stents at 6 months, revealing that OCT-guided PCI showed slightly fewer uncovered struts than angio-guided PCI, whereas no significant difference was observed in the rate of malapposition.^47^ As of this writing, definitive superiority of OCT-guided PCI over IVUS-guided PCI has not been demonstrated in prospective studies in terms of PCI optimization.

**Table 2:** Clinical Studies on OCT-Guided PCI

Study	N	Clinical Presentation	Design	Control	Endpoint	Results
ILUMIEN I^45^	418	SAP, NSTE-ACS	Observational	N/A	Change in strategy	55% of cases. Post-stent optimization in 27%
ILUMIEN II^25^	572	SAP, NSTE-ACS	Compared with historical control	IVUS-guided	Stent expansion	72.8% vs 70.6%, *P* = 0.29
OCTACS^47^	100	ACS	RCT (1:1)	Angio-guided	Uncovered struts at 6 mo Complete covered stent at 6 mo	4.3% vs 9.0%, *P* < 0.01 17.5% vs 2.2%, *P* = 0.02
OPINION^26^	829	SAP/UAP	RCT (1:1)	IVUS-guided	TVF at 1 y	5.2% vs 4.9%, noninferiority, *P* = 0.042
DOCTORS^28^	240	NSTE-ACS	RCT (1:1)	Angio-guided	FFR post PCI	0.94 vs 0.92, *P* = 0.005
ILUMIEN III^27^	450	SAP, NSTE-ACS	RCT (1:1:1)	IVUS-guided Angio-guided	Minimal stent area Procedural MACE requiring additional procedures	5.79 vs 5.89 vs 5.49 mm^2^, noninferiority to IVUS, *P* = 0.001 3% vs 1% vs 1%, *P* = 0.37
RENOVATE-COMPLEX-PCI^88^	1639	SAP, ACS	RCT (2:1) OCT or IVUS vs Angio	Angio-guided	TVF at 2 y	7.7% vs 12.3%, *P* = 0.008
ILUMIEN IV^89^	2,487	SAP, ACS	RCT (1:1)	Angio-guided	Post-PCI MSA TVF at 2 y	5.72 vs 5.36 mm^2^, *P* < 0.001 7.4% vs 8.2%, *P* = 0.45
OCTOBER^90^	1,201	Complex bifurcation	RCT (1:1)	Angio-guided	MACE at 2 y	10.1% vs 14.1%, *P* = 0.035
OCTIVUS trial^91^	2,000	SAP, NSTE-ACS	RCT (1:1)	IVUS-guided	TVF at 1 y	2.5% vs 3.1% Noninferiority, *P* < 0.001
COMBINE INTERVENE^a^ (NCT05333068)	1,222	SAP, ACS	RCT (1:1) FFR and OCT vs FFR	FFR-guided	MACE at 2 y	N/A

ACS = acute coronary syndrome; FFR = fractional flow reserve; IVUS = intravascular ultrasound; MACE = major adverse cardiac event(s); N/A = not available; NSTE-ACS = non-ST-elevation acute coronary syndrome; OCT = optical coherence tomography; PCI = percutaneous coronary intervention; RCT = randomized controlled trial; SAP = stable angina pectoris; TVF = target vessel failure; UAP = unstable angina pectoris.

a Ongoing study.

## OCT as a Research Tool

### Plaque erosion

**Figure 7:**
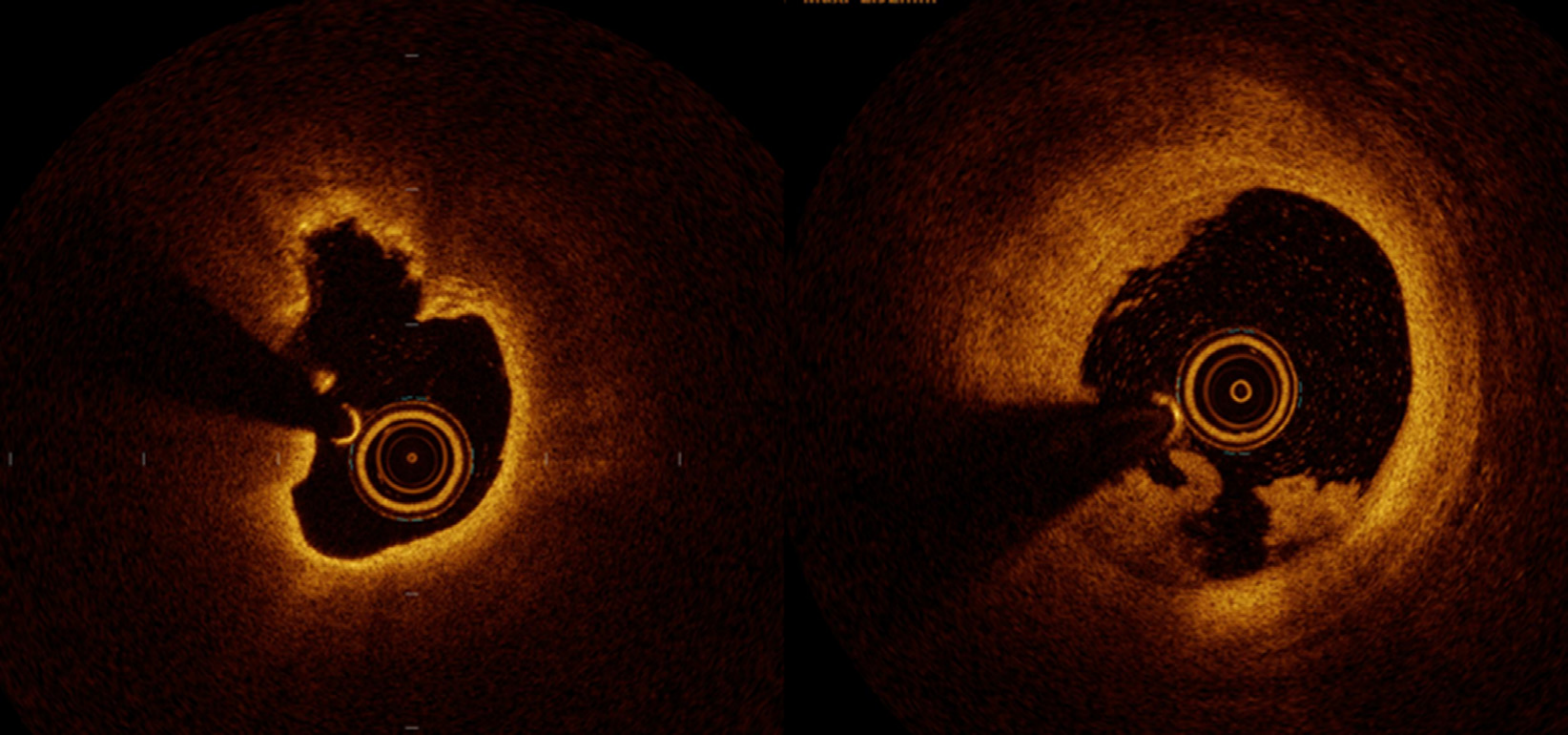
Ruptured Plaque and Plaque Erosion OCT provides a clear differentiation of plaque phenotypes at the culprit lesions of acute coronary syndrome. Plaque rupture is identified by a disrupted fibrous cap overlying lipid-rich plaque with or without cavity formation (left). Plaque erosion is characterized by intraluminal thrombus overlying fibrous or lipid plaque without fibrous cap disruption (right). Abbreviation as in Figure 1.

One of the most significant advantages of OCT is its ability to characterize plaque phenotype in the culprit lesions of ACS. A diagnostic algorithm for underlying plaque phenotypes, such as plaque rupture, plaque erosion, or calcified nodules was developed in 2013 using 128 patients with ACS from the MGH OCT registry.^48^ This study demonstrated that OCT could differentiate plaque phenotypes of the culprit lesions, showing a similar proportion of each plaque type compared with previous pathology studies.^49^^,^^50^ The diagnostic algorithm introduced in this study has been widely used for identification of OCT-defined plaque erosion, characterized by an intact fibrous cap without plaque rupture and intraluminal thrombus formation (Figure 7). Considering the nonocclusive thrombosis and smaller plaque burden with plaque erosion, several studies attempted conservative management without stent implantation for lesions with plaque erosion.^51^^,^^52^ The EROSION (Effective Anti-Thrombotic Therapy Without Stenting: Intravascular Optical Coherence Tomography-Based Management in Plaque Erosion) study, published in 2017, was a prospective observational study that investigated the feasibility of a conservative strategy with antithrombotic therapy for patients with ACS with OCT-defined plaque erosion in the culprit lesions. The study revealed a significant decrease in thrombus volume with antiplatelet therapy without stenting at the 1-month OCT follow-up, showing no adverse cardiovascular events in the 60 enrolled patients with erosion.^52^ The subsequent EROSION III study, which randomized 226 patients with STEMI into OCT-guided reperfusion and angio-guided reperfusion groups revealed that the OCT-guided reperfusion strategy resulted in fewer stent implantations than the angio-guided group; no significant difference was observed in the rate of adverse events.^53^ The EROSION study facilitated the application of OCT in patients with ACS, and investigators are currently exploring a potential paradigm shift in ACS management with the identification of plaque erosion and conservative management without coronary stenting in certain subsets of patients. These preliminary findings should be validated in large-scale randomized trials in the future. Since the feasibility of the OCT-derived diagnostic algorithm of plaque erosion was reported, research interest in the clinical and morphological characteristics of plaque erosion has grown. Yamamoto et al^54^ investigated 1,241 patients with ACS undergoing OCT examination of culprit lesions, identifying plaque erosion in 38.4% of all patients with ACS, with NSTE-ACS showing a greater prevalence of erosion compared with STEMI (47.9% vs 29.8%; *P* < 0.001). This study found that age <68 years, anterior ischemia, no diabetes mellitus, a hemoglobin of >15.0 g/dL, and normal renal function were independent predictors of OCT-derived erosion.^54^ The predictors of OCT-derived plaque erosion at the culprit lesions in patients with STEMI showed that younger age, current smoking, absence of coronary risk factors, larger vessel size, and the spatial location with close proximity to a bifurcation were independent predictors of erosion.^55^ However, differentiating plaque erosion from plaque rupture using clinical characteristics alone remains challenging. Future studies should aim to identify additional diagnostic methods for the noninvasive diagnosis of erosion.

### Healed coronary plaque

**Figure 8:**
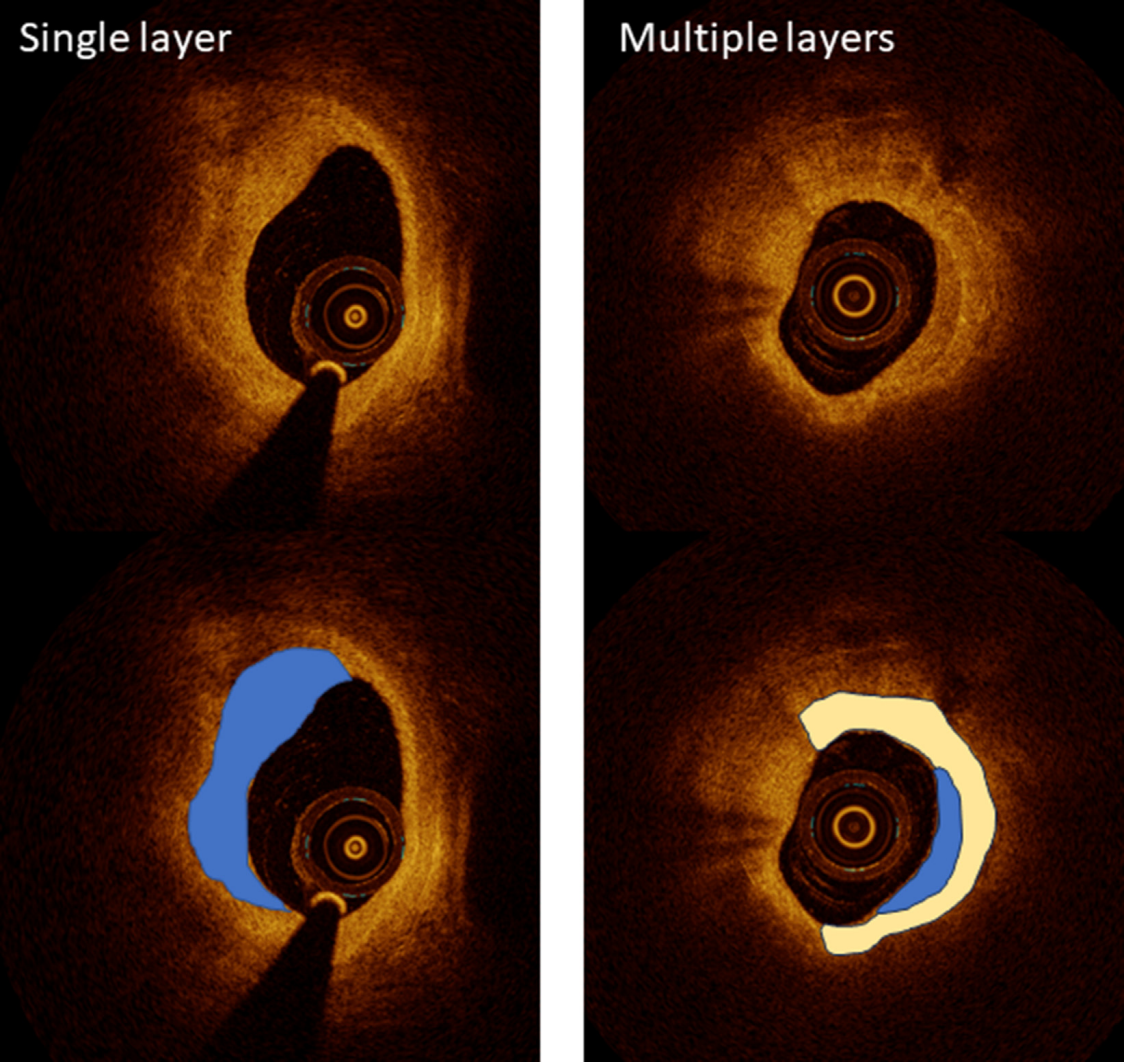
Layered Plaque Atherosclerotic plaque sometimes shows a layered appearance which is reported to represent healed coronary plaque. A crescent shaped layer of different optical density with a clear demarcation from underlying components exists adjacent to the lumen (left). Tight stenosis may harbor multiple layers (right).

Coronary thrombosis after plaque rupture or erosion does not always cause clinical symptoms. Even after plaque rupture, an occlusive thrombus may not develop in the coronary artery, which may heal with endogenous antithrombotic mechanisms. Previous pathology studies have indeed found multiple healed sites from previous ruptures underneath the acute rupture site, evident as layered tissues overlying a necrotic core.^56^ OCT is able to identify the multiple layers of healed coronary plaque (Figure 8), which was validated with pathology examination by Shimokado et al.^57^ As suggested by previous pathology studies,^56^ plaque disruption followed by organization of residual thrombus may contribute to rapid progression of the lesion. This progression results in the formation of layered pattern of coronary plaque on OCT.^58^^,^^59^ Thus, the OCT-defined healed coronary plaque, characterized by the layered plaque, represents indicators of previous plaque destabilization. It has been reported that the healed coronary plaque at the culprit lesions was associated with plaque vulnerability at both culprit and nonculprit sites. Usui et al^60^ reported that untreated healed coronary plaque was associated with future adverse events, predominantly ischemia-driven revascularization. As mentioned elsewhere in this article, because healed coronary plaque can be detected by OCT, it has gained research attention as a potential marker of plaque vulnerability.

### Calcified plaque in ACS: A new classification

**Figure 9:**
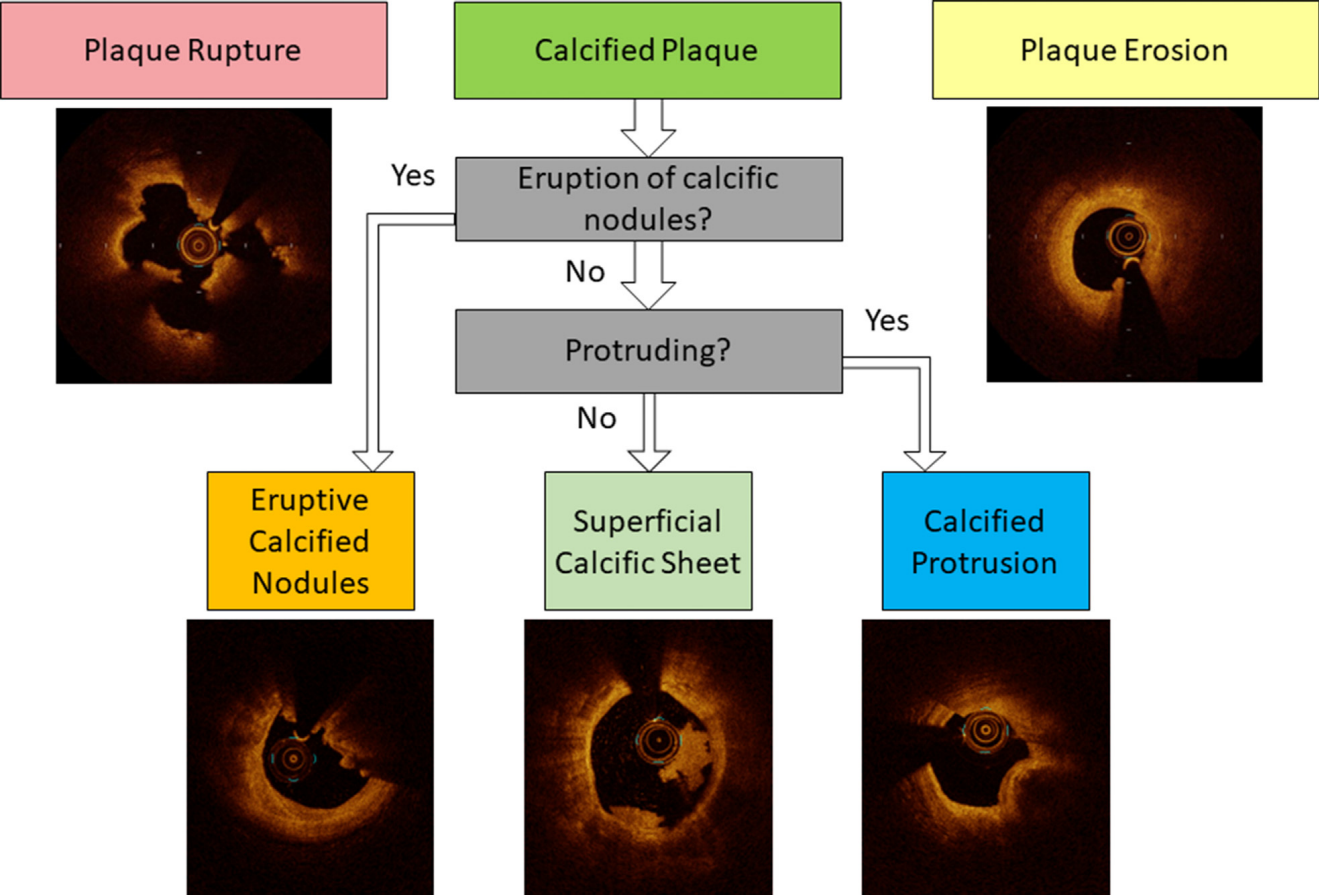
New Classification for Calcified Culprit Lesions in ACS Calcified plaque can be a cause of ACS, after plaque rupture and plaque erosion, of which 3 subtypes can be identified by OCT: eruptive calcified nodules, superficial calcific sheet, and calcified protrusion. ACEs = acute coronary syndrome; other abbreviation as in Figure 1.

OCT has enriched our understanding of coronary calcification. When compared with other intravascular imaging modalities, OCT is superior in the assessment of the morphological characteristics of vascular calcification. Previous OCT studies investigated the morphological characteristics of ACS culprit lesions and identified calcified nodules as one of the causes of coronary thrombosis, corroborating findings from pathology studies. Additionally, as more clinical data accumulated, the morphological variety of calcification at the culprit lesions of ACS have been noted. Sugiyama et al^61^ reported that calcified plaques represented 141 (12.7%) of the culprit lesions in 1,241 patients with ACS. More important, they found 3 distinct subtypes: eruptive calcified nodules (which corresponded with pathologically defined calcified nodules), superficial calcific sheets (which displayed a sheet-like appearance of a superficial calcific plate without erupted nodules or protruding masses into the lumen), and calcified protrusions (which corresponded with pathologically defined nodular calcification overlaid by smooth fibrous tissue) (Figure 9). Interestingly, only one-fourth of these were eruptive calcified nodules; the majority were superficial calcific sheets. Thus, close observation of ACS culprit lesions using OCT led to discovery of 3 subtypes of calcified plaques in vivo and the development of a novel classification that extends beyond existing knowledge based on autopsy studies.

### Vulnerable plaque research

The term vulnerable plaque was introduced in 1989 by Muller et al^62^ to describe 2 conditions of coronary plaque: vulnerable plaque, which refers to a plaque susceptible to rupture triggered by various stimuli such as circadian rhythm, and nonvulnerable plaque. The initial definition of vulnerable plaque focused on plaques that were prone to rupture, potentially leading to coronary thrombosis and the onset of ACS. However, owing to the low incidence of hard endpoints such as AMI and SCD, clinical trials included soft endpoints such as revascularization. Hence, we should be cautious in interpreting the results of clinical studies. In 2011, the PROSPECT (Providing Regional Observations to Study Predictors of Events in the Coronary Tree) study identified virtual histology IVUS-derived vulnerable plaque features, including virtual histology-derived TCFA, large plaque burden, and small lumen area in the nonculprit lesions of patients with ACS using virtual histology IVUS examinatoin.^63^ After this landmark study, several OCT studies aimed to identify vulnerable features using OCT (Table 3). The MGH group conducted a retrospective analysis of 1,474 patients and reported that lipid-rich plaque identified in the nonculprit regions of the target vessel was associated with subsequent adverse events at 4 years.^64^ The CLIMA (Relationship Between Coronary Plaque Morphology of Left Anterior Descending Artery and Long Term Clinical Outcome) study reported that a minimal lumen area of <3.5 mm^2^, fibrous cap thickness of <75 μm, lipid arc of >180°, and OCT-defined macrophages were associated with an increased risk of the composite of cardiac death and target segment MI.^65^ The COMBINE OCT-FFR (Combined Optical Coherence Tomography Morphologic and Fractional Flow Reserve Hemodynamic Assessment of Non-Culprit Lesions to Better Predict Adverse Event Outcomes in Diabetes Mellitus Patients) trial demonstrated that, among lesions with a negative FFR (>0.80), TCFA more frequently developed adverse cardiac events.^65^ More recently, Jiang et al^66^ investigated 883 patients with AMI who underwent OCT examination of all 3 main epicardial coronary arteries after primary PCI. They reported that TCFA and a minimum lumen area of <3.5 mm^2^ were predictive of adverse cardiac events in both lesion-level and patient-level analyses, and, when these 2 factors were present, the HR for lesion-specific analysis was 15.5 (95% CI: 6.89-34.89).^66^ These OCT-based studies consistently showed that TCFA/lipid-rich plaque with a small minimum lumen area was the hallmark of adverse events. Vulnerable features have been identified by various imaging modalities, which typically include lipid-laden plaque characteristics, a small lumen area, and a large plaque burden. However, in all studies, the primary endpoint was driven by unplanned revascularization and not by a hard endpoint. The CLIMA study was an outlier. Furthermore, we need to consider the diverse mechanisms of coronary thrombosis, including plaque erosion and calcified nodules. Currently, no diagnostic method is available to detect plaques prone to endothelial denudation and occlusive thrombus formation leading to plaque erosion. Considering all these issues, the ability to predict all future cardiovascular events with intravascular imaging modalities including OCT may be in the distant future.^67^

**Table 3:** Vulnerable Plaque Studies/ACS Studies

Study	N	Clinical Presentation	Design	Groups	Endpoint	Results
Lei Xing et al^64^	1,474 lesions	All	Observational	Lipid rich vs non lipid rich	MACE (CV death, AMI, ischemia-driven revascularization)	7.2% vs 2.6% at 4 years, *P* = 0.033
CLIMA^92^	1,003, 1,776 plaques	All with untreated LAD	Observational	N/A	CV death or target vessel MI at 1 year	MLA <3.5 mm^2^, FCT <75 μm, lipid-arc >180, and macrophages were independent risks
EROSION^52^	60	ACS with erosion	Observational	N/A	>50% reduction of thrombus at 1 month after conservative management	47 patients (78.3%) met the criteria
CLI-OPCI II^31^	1,002	All	Observational	N/A	MACE (death, MI, TLR)	MLA <4.5 mm^2^, dissection >200 μm, and reference LA <4.5 mm^2^ were independent predictors
CLI-OPCI ACS^34^	507	ACS	Observational	N/A	MACE (death, MI, TLR)	MLA <4.5 mm^2^, protrusion, dissection >200 μm, and reference LA <4.5 mm^2^ were independent predictors
HUYGENS^93^	161	NSTEMI	RCT	Evolocumab vs placebo	Change in FCT and lipid arc at 1 year	+43 μm vs +22 μm, *P* = 0.015 –58° vs –31°, *P* = 0.04
PACMAN-AMI^94^	300	AMI	RCT	Alirocumab vs placebo	Change in FCT at 1year, as the 2˚ endpoint	+62.4 μm vs +28.2 μm, *P* = 0.01
COMBINE OCT-FFR^65^	390	All with negative FFR	Observational	TCFA vs non-TCFA	MACE (CV death target-vessel MI, TLR, UAP hospitalization) at 18 months	13.3% vs 3.1%, *P* < 0.0001

AMI = acute myocardial infarction; CV = cardiovascular; FCT = fibrous cap thickness; LA = lumen area; MI = myocardial infarction; MLA = minimum lumen area; TCFA = thin-cap fibroatheroma; TLR = target lesion revascularization; other abbreviations as in Table 2.

### Vascular biology research

One of the most significant contributions of OCT has been its role in enhancing our understanding of atherosclerosis. Because intravascular OCT provides high-resolution images of vessel microstructures close to the lumen, it has been extensively used to examine various aspects of vascular biology in coronary arteries, particularly in ACS. In 2012, Kato et al^68^ investigated nonculprit plaque characteristics in 104 patients with ACS and non-ACS and reported that nonculprit lesions in patients with ACS had larger lipid indices (defined as the average lipid arc multiplied by lipid length), and more prevalent TCFAs compared with those in stable patients. This finding supports the important concept of panvascular inflammation in patients with ACS.^69^^,^^70^ In line with this finding, when patients with ACS were divided into those with plaque rupture at the culprit lesion and those without, nonculprit plaques showed rupture in 26% of patients with plaque rupture at the culprit lesion, whereas no plaque rupture was observed in the counterpart (26% and 0%; *P* = 0.02).^70^ These results underscore the idea that ACS is a panvascular process with local manifestations and that prevention and treatment should be patient focused rather than plaque focused. OCT has been used to evaluate the morphological features of the culprit lesions of ACS as described,^48^ and numerous studies have shed light on the panvascular nature of atherosclerosis. Araki et al^71^ conducted a comprehensive analysis of the distribution of coronary plaques with different phenotypes using 3-vessel OCT data, discovering that TCFA was particularly clustered in the proximal segment of the left anterior descending artery (LAD) in patients with ACS. In contrast, fibrous plaques were evenly distributed throughout the coronary trees. This study provided new insights into the in vivo distribution of various types of coronary plaques and their associations with clinical presentations.^71^

### Neoatherosclerosis

**Figure 10:**
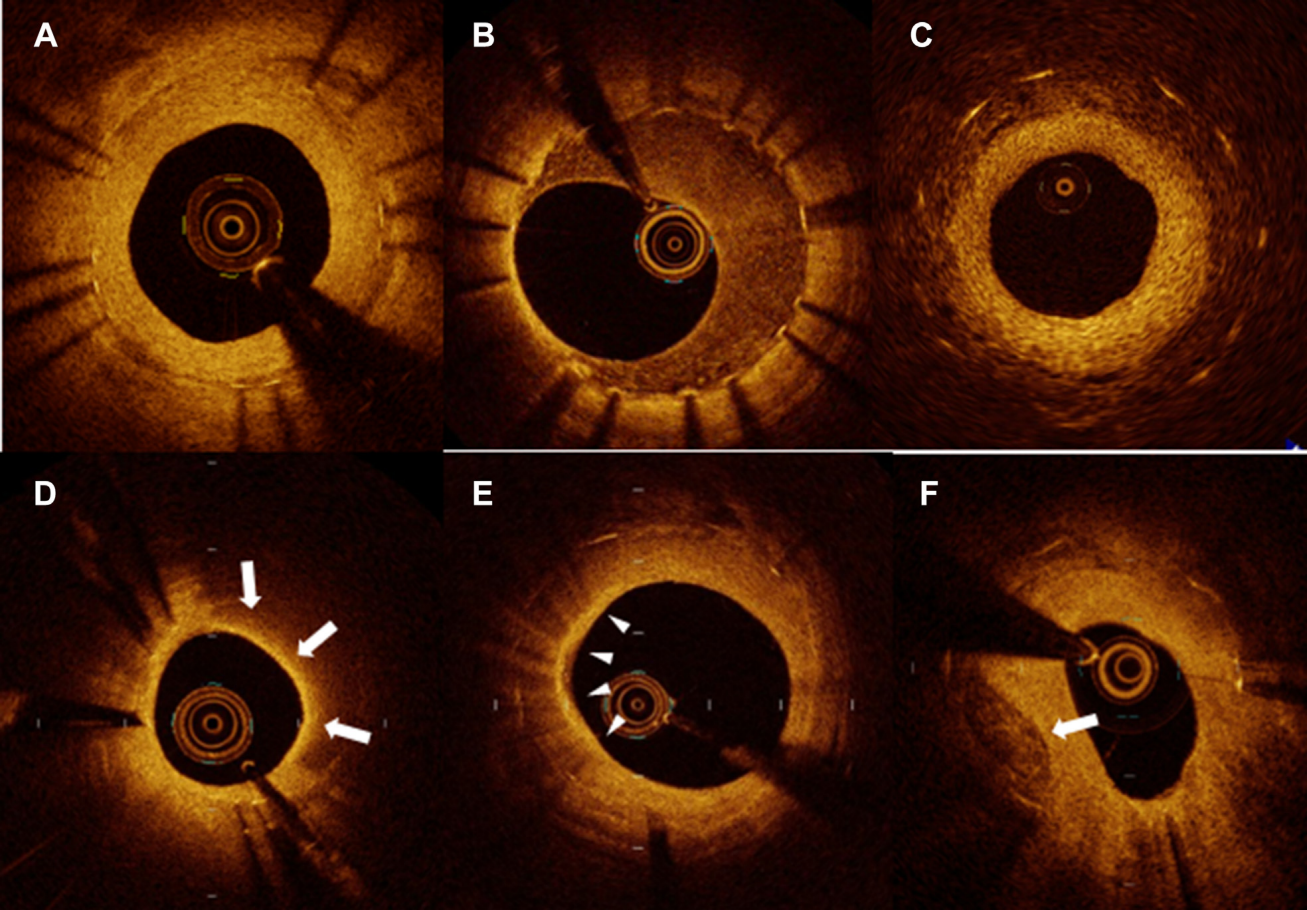
Neointimal Patterns Within Stents Observed by OCT (A) Homogeneous neointima shows a uniform optical property inside the stent. (B) Heterogeneous neointima is characterized by a low backscattering tissue containing focal bright spots inside the tissue. (C) Layered neointima shows concentric layers with different tissue properties: adluminal high-intensity and abluminal low-intensity layers. (D) Lipid-laden neointima is identified by a signal poor region with diffuse border overlying signal-rich layer representing a fibrous cap (white arrows). Signal attenuation precludes visualization of stent strut. (E) Macrophage accumulation appears as signal-rich, distinct, or confluent punctate regions that exceed the intensity of background speckle noise (arrowheads). The strong attenuating properties cause the casting of a laterally sharp shadow and rapid change in appearance from frame to frame. (F) Calcified neointima is characterized by a signal-poor region with sharp border (white arrow).

OCT has been used to evaluate the underlying mechanisms of stent failure in previously implanted stents, as IVUS was recommended by earlier versions of American and European guidelines.^15^ Gonzalo et al^72^ reported in 2009 that OCT can depict various patterns of neointima within stents with restenosis, which was not provided by IVUS or other imaging modalities (Figure 10). In addition to homogeneous, heterogeneous, or layered pattern neointima, Takano et al^73^ reported the development of lipid-laden neointima observed by OCT within stents. Notably, lipid-laden neointima was more frequently observed in bare-metal stents implanted >5 years previously as compared with younger bare-metal stents implanted within 6 months (67% and 0%; *P* < 0.01). After this report by Takano et al,^73^ the development of newly formed lipid-laden neointima or calcified neointima within stents was defined as neoatherosclerosis^74^ to align with the pathological concept.^75^ Expanding on the OCT examination data from the MGH OCT registry, we evaluated both bare-metal stents and drug-eluting stents, finding that drug-eluting stents, stent age >48 months, chronic kidney disease, absence of angiotensin II receptor blocker, age >65 years, and current smoking were independent predictors of neoatherosclerosis.^76^

### Very late stent thrombosis

Stent thrombosis is an unresolved issue in the treatment of coronary artery disease with PCI. When the first-generation drug-eluting stent was introduced early this century, it seemed promising in overcoming coronary artery disease, exhibiting a significantly lower restenosis rate, which had been a major obstacle in PCI. However, subsequent studies raised concerns about the safety of drug-eluting stents, noting a potentially increased risk of stent thrombosis that could persist for years, as the risk for very late stent thrombosis. OCT examinations contributed to elucidate the mechanisms of very late stent thrombosis in vivo. A study by Souteyrand et al,^40^ which observed 120 stent thrombosis cases using OCT, revealed that acute or subacute stent thrombosis was predominantly attributable to malapposition or underexpansion. In contrast, stents with very late stent thrombosis frequently showed neoatherosclerosis within the stent.^40^ Taniwaki et al^39^ also reported a consistent result showing that approximately 30% of the stents with VLST harbored neoatherosclerosis. Presently, OCT is widely recognized as the imaging modality of choice for investigating the mechanisms of stent thrombosis in vivo.

### MI with nonobstructive coronary arteries

Recent years have witnessed increased clinical interest in MI with nonobstructive coronary arteries (MINOCA) as a non-negligible proportion of the clinical presentation of AMI. As described previously, angiography may not necessarily detect the cause of coronary thrombosis owing to its limited resolution. OCT, given the higher resolution images, may identify the microstructures of coronary lesions responsible for myocardial injury. such as intraluminal thrombus, plaque rupture, plaque erosion, calcified nodule, intraplaque hemorrhage, layered plaque, or spontaneous coronary dissections. It was reported that approximately 25% of patients with MINOCA harbored abnormal OCT findings.^77^ Usui et al^78^ investigated the characteristics of MINOCA diagnosed with a combination of angiography and cardiac magnetic resonance imaging in comparison with AMI with obstructive coronary lesions. They reported that the culprit lesions of MINOCA showed more frequent intraplaque hemorrhage and layered plaque, compared with AMI with obstructive coronary arteries. Moreover, a recent study has shown that the baseline OCT findings in patients with MINOCA was significantly associated with subsequent clinical outcomes.^24^ These studies have suggested the potential role of OCT in the diagnosis and management of patients with MINOCA.

## Perspective

As described, intravascular OCT, with its superior spatial resolution and capability for plaque characterization, has made significant contributions to cardiovascular medicine, mainly in optimizing PCI procedures and improving our understanding of vascular biology. PCI optimization has been largely dependent on the software and hardware for OCT imaging and has been driven by industry. Although vascular biology research emerged in the early 2010s, its popularity has decreased and is now limited to a small number of research groups. In daily practice, the penetration rate of OCT has not surpassed other imaging modalities such as IVUS examination. To increase the widespread use of OCT imaging and establish it as an essential modality in clinical practice, several issues must be addressed. One issue lies in the interpretation of OCT images, which requires additional training and experience. Artificial intelligence (AI) seems to be a promising solution and has been the focus of intensive research in recent years. Numerous groups have attempted to establish deep learning models for accurately diagnosing specific plaque types using AI.^79^, ^80^, ^81^ Park et al^79^ reported the extremely high diagnostic accuracy of a novel “transformer-based” deep learning model that takes into consideration adjacent frames of the selected image for diagnosis, imitating expert readers evaluating consecutive OCT images frame by frame when assessing plaque phenotypes. For diagnosing plaque erosion, the transformer-based deep learning model demonstrated an excellent C-statistic of 0.94, compared with a convolutional neural networks model with a C-statistic of 0.85.^79^ If implemented in clinical practice, this model could enable rapid, accurate, and objective diagnosis of underlying plaque characteristics in ACS, further facilitating tailored therapeutic strategies based on plaque phenotypes, as described elsewhere in this article. The latest OCT console produced by Abbott Medical (Ultreo, Abbott Medical) incorporates an AI-based algorithm for delineating calcification, automatically displaying the angle and thickness of calcium within the plaque. Future research efforts should aim to develop prediction models for subsequent outcomes rather than human diagnosis. Furthermore, because the primary purpose of AI-based OCT imaging is to provide accurate and objective information to the operator, implementation into OCT equipment is essential for its use in daily practice.

Another issue to be addressed for the future of intravascular OCT is procedural limitations, such as the need to eliminate blood from the vessel lumen, difficulty with ostial lesions, and an inability to evaluate the entire vessel wall in large vessels. Given the nature of OCT technology, many of these issues may not be overcome by themselves. However, research efforts have been made to complement OCT imaging with IVUS examination. As several previous studies have reported important aspects of vascular biology by using both OCT and IVUS imaging in the same lesions,^82^^,^^83^ combining these modalities enables a detailed in vivo assessment of the entire vessel. Because using 2 imaging catheters is costly and not economically viable, multiple groups have been trying to develop combined OCT and IVUS catheters. To date, 2 OCT-IVUS combined catheters have been used in humans^84^^,^^85^: the Novasight Hybrid System (Conavi Medical Inc) and the Dural Sensor IVUS-OCT (Terumo), both of which are still in the early stages of investigation in limited countries before being launched in the global market. The challenges in combining these 2 different modalities lie in minimizing catheter size, coregistering the IVUS and OCT images, and adjusting pull-back speeds. The 2 developers used different strategies to address these challenges. Conavi positioned the OCT lens and IVUS transducer colinearly and overlapping, which enabled accurate coregistration. In the Terumo system, the optical lens and IVUS transducer are located side by side, allowing for a fast and long pull-back in a single session. With the advent of IVUS-OCT combined catheters, a wealth of information can be acquired by imaging modalities, potentially facilitating imaging-guided PCI and expanding our understanding of vascular biology. In addition to these advancements, consistent research efforts have been made in various directions. Although current OCT imaging provides higher resolution images compared with previous imaging modalities, there are limitations in spatial resolution. For example, OCT is not capable of visualizing a single layer of endothelial cells, which could lead to a definitive diagnosis of erosive plaque. Micro OCT (μOCT) is an advanced OCT system using a shorter light wavelength centered at 800 μm, providing extremely high resolution of 1 to 3 μm and enabling the visualization of individual cells within the vessel wall.^86^ However, μOCT has limitations, including limited tissue penetration and image acquisition rate. Shorter wavelengths of near-infrared light may improve image resolution at the expense of signal penetration depth. Additionally, because higher resolution images contain more data and place a greater load on the system, the image acquisition rate of μOCT is not sufficient for clinical use. Owing to these unresolved limitations, μOCT has not been tested in humans.

## Conclusions

Since the invention of OCT at MIT and the first-in-man study of intracoronary OCT >2 decades ago, this technology has made significant strides in cardiovascular medicine. With its high spatial resolution and detailed plaque characterization, OCT has primarily impacted PCI optimization and vascular biology research. Although not currently an essential mode of coronary imaging in clinical practice, history moves forward. The integration of AI and new technologies, along with the development of next-generation OCT software and hardware, promises to bring us closer to the ideal optimization of PCI and a deeper understanding of vascular biology. These advancements have the potential to improve both the prevention and outcomes of coronary artery disease in the future.

## Funding Support and Author Disclosures

Dr Jang received an educational grant from Abbott Medical; and served on the clinical end point committee for Svelte Medical System. Dr Yonetsu has reported that he has no relationships relevant to the contents of this paper to disclose.
